# Skin as a Reflection of Gut Health: An Overview of Dermatological Manifestations in Primary Neoplastic and Autoimmune Gastrointestinal Disorders

**DOI:** 10.7759/cureus.71313

**Published:** 2024-10-12

**Authors:** Fatima Hajj, Vaishnavi Singh, Nourhane Al Akoum, Nikita Patil, Farah N Ahmad, Andres Chuecos, Pranavi Vemana, Gilberto González, Yahya Makkieh, Douaa Al Farou, Janisha Paul, Humza F Siddiqui

**Affiliations:** 1 College of Medicine, Lebanese University Faculty of Medicine, Beirut, LBN; 2 Internal Medicine, Bhaskar Medical College, Hyderabad, IND; 3 College of Medicine, Beirut Arab University, Beirut, LBN; 4 Reproductive Medicine, Cryo Mediferti LLP, Mumbai, IND; 5 Family Medicine, Dubai Health, Dubai, ARE; 6 General Medicine, University of Los Andes, Mérida, VEN; 7 Medicine, GITAM Institute of Medical Sciences and Research, Visakhapatnam, IND; 8 College of Medicine, Monterrey Institute of Technology and Higher Education, San Pedro Garza García, MEX; 9 General Practice, Beirut Arab University, Beirut, LBN; 10 Medicine, Near East University, Nicosia, CYP; 11 Medicine, Punjab Institute of Medical Sciences, Jalandhar, IND; 12 Internal Medicine, Jinnah Postgraduate Medical Centre, Karachi, PAK

**Keywords:** autoimmune gastrointestinal diseases, autoimmune hepatitis, celiac disease, dermatitis herpetiformis, dermatological manifestations, gastrointestinal neoplasms, inflammatory bowel disease, leser-trélat sign, primary biliary cholangitis, primary sclerosing cholangitis

## Abstract

Gastrointestinal (GI) diseases can present with several extraintestinal manifestations, and cutaneous signs and symptoms are most frequent. Although conventionally GI and skin are considered two entirely separate organ systems, they are closely correlated in origin. An increasing amount of data highlights the complex relationship between GI and dermatological conditions. This review article aims to particularly explore the clinical correlation between neoplastic and autoimmune GI disorders and skin manifestations, which serve as clinical indicators of these diseases. Neoplastic diseases including pancreatic cancer, gastric adenocarcinoma, Muir-Torre syndrome, carcinoid syndrome, and malignant and benign colorectal polyposis syndromes can be accompanied by skin conditions like pancreatic panniculitis, acanthosis nigricans, keratoacanthomas, necrolytic migratory erythema, melanotic macules, oral papillomas and osteomas, respectively. Autoimmune diseases including celiac disease, autoimmune liver conditions and inflammatory bowel disease (IBD) have been linked to dermatological manifestations such as xanthomas, morphea, psoriasis, dermatitis herpetiformis, erythema nodosum and epidermolysis bullosa acquisita. The skin manifestations can precede the GI symptoms and indicate the severity of the underlying condition, serving as a significant diagnostic marker earlier in the disease. Resolution of skin findings can also indicate the efficacy of treatment strategies and provide prognostic utility. Further research is essential to outline the underlying mechanisms linking dermatological and GI diseases and there is an immediate need for collaborative efforts between dermatologists and gastroenterologists.

## Introduction and background

The gastrointestinal-dermatological axis refers to a genetic, neoplastic and/or autoimmune correlation between the alimentary system and the skin which is the target of this study [1]. A careful understanding of a strong association between these two systems can reveal how a disease in one system can manifest symptoms into the other. Of note, dermatological signs can be an alarming finding and herald the clinical symptoms of an occult gastrointestinal (GI) disease [2]. The coexistence of GI and cutaneous findings is not a matter of coincidence. Instead, it is the end result of a shared genetic, histological and clinical background. For instance, GI inflammatory disorders may accompany psoriasis, which is an immune-mediated chronic inflammatory condition and has a systemic involvement. This is explained by the fact that psoriasis has common pathogenic pathways with GI immune-mediated chronic inflammatory disorders namely celiac, inflammatory bowel disease (IBD) and nonalcoholic fatty liver disease. The identification of shared susceptibility loci and DNA polymorphisms has confirmed this correlation at a genetic level [3-5].

A thorough recognition of this correlation is the need of the hour to alert an attentive physician, to provide an accurate diagnosis of occult alimentary tract diseases and to offer appropriate management plans. What is also a key behind studying this axis is screening of family relatives and early detection of hereditary diseases. The co-occurrence of findings common between GI and dermatological systems highlights the presence of a genetic syndrome and hereditary malignancies. For instance, familial adenomatous polyposis (FAP), which causes thousands of polyps in the colon, is the end product of a germline mutation affecting the adenomatous polyposis coli (APC) gene with an autosomal dominant inheritance pattern [6]. A patient with dermoid cysts, lipomas, and desmoid tumors should be evaluated for polyposis of the colon [7]. Early recognition of this condition is essential for planning prophylactic colectomy and screening of family relatives. Understanding that these disorders might coexist or predispose relatives is a key to raising a high index of suspicion leading to an early diagnosis and a decrease in morbidity with appropriate prophylactic treatment and screening measures [3]. One of the well-known GI neoplastic diseases is colorectal cancer accounting for 900,000 yearly cases around the globe [8]. Skin manifestations of this heritable condition play a fundamental role in disclosing occult colon cancers, thus decreasing morbidity and mortality. IBD constituting Crohn’s disease and ulcerative colitis (UC) has several extraintestinal manifestations, including the skin. Cutaneous findings including erythema nodosum (EN), pyoderma gangrenosum (PG), and aphthous stomatitis can occur before, after or simultaneously with intestinal disease, thus impacting the morbidity and well-being of the affected patient [2,9,10].

This review aims to disclose the correlation between the skin and the gut. This is achieved by describing common GI diseases, including autoimmune and neoplastic conditions and their specific cutaneous manifestations. It also highlights the importance of understanding the GI-dermatological axis for an early diagnosis and a decrease in morbidity and mortality. It highlights that the interaction of the integumentary and alimentary systems is an area of clinical inquisitiveness.

## Review

Neoplastic diseases

The intricate connection between skin manifestations and underlying systemic diseases has gained recognition recently. In the realm of GI malignancies, specific skin findings can be crucial for early detection and treatment. Recent research has highlighted the significance of dermatological manifestations in these conditions, paving the way for a more integrated approach to diagnosis and management.

Hereditary GI polyposis syndromes include adenomatous and hamartomatous polyposis syndromes (HPS). The latter includes disorders such as juvenile polyposis syndrome, Peutz-Jeghers syndrome (PJS) and Cowden syndrome (CS) amongst others which account for around 5-10% of all colorectal cancers [11].

PJS

PJS is an inherited autosomal dominant disorder involving germline mutations in the STK11/ LKB1 gene. It is characterized by hamartomatous polyps spread along the GI tract, particularly the small intestine and colon causing bowel occlusions, bleeding, and intussusception. Affected individuals have an increased susceptibility to developing GI malignancies like colorectal, gastric and pancreatic cancer, particularly in the small intestine during the third decade of life [12].

Characteristic skin derangements in the form of 1-5 mm melanotic macules over the labial mucosa, palate and tongue before the first decade of life are essential for diagnosing this multisystemic condition. Rahvar and Kerstetter state that the presence of hyperpigmented macules and cutaneous lesions usually precedes GI symptoms. Most of these lesions fade after puberty, unlike the oro-buccal mucosal pigmentation that persists lifelong [2].

The presence of abdominal discomfort along with the severity of dermatological findings in a patient with oral hyperpigmentation is suggestive of PJS-associated polyps [12]. Sandru et al. further suggested that baseline surveillance with esophagogastroduodenoscopy should be done from eight years of age and colonoscopy after the age of 50, once every two years in case of the above-mentioned symptoms. A study recommends that initial genetic screening should be done in asymptomatic children and adolescents with lip and mucosal freckling, which is suggestive of PJS as per the European Society for Pediatric Gastroenterology Hepatology and Nutrition [13].

CS

CS is a rare hereditary cancer predisposition syndrome that is caused by a PTEN gene mutation, associated with a high risk for malignancies of the colon, breast, thyroid, kidney, endometrium, and skin. It is characterized primarily by malignancies, GI hamartomas, diffuse glycogenic acanthosis in the esophagus, macrocephaly and multiple mucocutaneous lesions such as trichilemmomas, oral papillomas, acral keratosis, lipomas and maculopapular pigmentation of the glans penis, which usually appear by 30 years of age [14]. In addition, multiple cases have reported cutaneous lesions such as xanthomas, vitiligo, acral and periorificial lentigines, café au lait spots, hemangiomas and occasionally skin tags, reported to be found in 16% of CS patients [15]. These features develop in over 80% of CS patients prior to the neoplastic changes; hence, an early dermatological examination should be implemented from the late teens [16].

Takayama et al. conducted a study that reported that mucocutaneous neuromas were seen in five out of 12 cases involving children under 10 years of age, providing a hint to the childhood diagnosis of CS. It further states that histologically, hamartomatous polyps are the most common amongst colorectal polyps in CS patients with the lifetime risk of colorectal cancer being 9-16%, which is significantly more than that in healthy individuals [14].

Lee et al. presented a case report of a 21-year-old female patient with CS, who exhibited dermatological manifestations of acanthosis nigricans and multiple small, white papules on her palms and neck, with several pedunculated lesions on her left axilla. Histopathology of a punch biopsy of the left palm showed orthokeratosis and hyperkeratosis revealing it to be palmoplantar keratosis, which has a prevalence rate of 10.2-82% in PTEN-positive CS patients [15,17].

Another case report described a case of a 53-year-old female, diagnosed with CS after a thorough analysis of her oro-facial manifestations, which constituted adenoid facies and numerous sessile, smooth, papular lesions of less than 4mm size spread over the buccal mucosa, alveolar ridges, tongue and both the oral commissures in a cobblestone manner [18]. This further highlights the need for dermatologists to recognize the variety of oral manifestations of CS for timely diagnosis.

Muir-Torre Syndrome (MTS)

MTS, a phenotypic variant of Lynch syndrome, is a rare hereditary cancer syndrome characterized by sebaceous tumors and visceral malignancies. It presents a fresh understanding of how genetics contributes to skin problems in people with systemic diseases. A study emphasized the diagnostic significance of sebaceous tumors and keratoacanthomas in MTS, often being the earliest signs of the disease [19]. This finding highlights the importance of thorough dermatological examination in individuals with a personal or family history of MTS or colorectal cancer, as early detection of these cutaneous tumors can trigger prompt genetic counseling, cancer screening and preventive measures.

Carcinoid Syndrome

Carcinoid syndrome is a rare condition associated with neuroendocrine tumors (NETs) and primarily affects patients with liver metastases of GI NETs, extra-GI NETs in areas like ovaries, testis, lungs/bronchus, or those with extensive involvement in midgut NETs. Common clinical features include diarrhea (60-80%), abdominal pain (40%), telangiectasia (25%), vascular heart disease (20%) and intermittent bronchial wheezing (<10%) [20]. Skin manifestations are a key diagnostic factor that includes flushing with 90% prevalence and pellagra skin symptoms exhibiting edema, pruritis and blisters are found in 20% of the patients. Other common dermatological symptoms include necrolytic migratory erythema, tongue inflammation, angular cheilitis and scleroderma-like features without Raynaud’s phenomenon [21]. The exact prevalence of carcinoid syndrome is still uncertain, but it has been documented to happen in almost 20% of individuals identified with NET. For diagnosis, imaging remains a key factor in the diagnosis of NETs, and given its high sensitivity and specificity, positron emission tomography (PET) and computed tomography (CT) should be the preferred imaging modality [22]. Costa et al. presented a case of a 66-year-old woman presenting with chronic diarrhea, who was diagnosed with carcinoid syndrome on liver biopsy. The author suggested that the occurrence of venous telangiectasia on the skin was the major diagnostic clue for the disease [23]. Ferreira et al. reported a rare case with ileal NET. The patient initially presented with scleroderma-like cutaneous manifestations, particularly widespread in the lower limbs, with no associated Raynaud’s phenomenon and the lesions disappeared after the treatment of the primary malignancy [24].

Gardner Syndrome

Gardner syndrome is part of the spectrum of FAP syndromes. It is an autosomal dominant genetic disorder resulting from mutations in the APC gene. The wide array of symptoms ranges from multiple colorectal adenomas to various types of extracolonic manifestations [25]. In the syndrome, dermatological features are particularly prominent in diagnosis and management.

Gardner syndrome, along with its typical colonic manifestations, has several cutaneous features that can act as valuable diagnostic and prognostic indicators. For instance, epidermoid cysts are skin lesions that are very common in Gardner syndrome. They usually appear on the face, scalp and trunk, even in early adulthood or adolescence. The cysts should be monitored through regular dermatological evaluation [26]. Additionally, fibromas also referred to as dermatofibromas, benign skin tumors are frequently observed in Gardner syndrome. They present as small, firm nodules, generally located on the extremities and trunk [27]. Moreover, desmoid tumors, although histologically benign, are aggressive and occur in almost all tissues of the body, with a special predilection for the abdominal wall. They are especially significant in Gardner syndrome and may present significant management challenges due to their tendency to be locally invasive and recurrent [28]. Lastly, osteomas are benign bone tumors that are fairly frequently described in Gardner syndrome. Typically, the mandible and skull are primarily affected by these tumors. They are largely asymptomatic but can be diagnosed with the aid of imaging studies [29].

In Gardner syndrome, skin lesions such as epidermoid cysts and fibromas are usually among the earliest indicators of the disease and can assist in its early detection. Clinical cases report a 40-year-old man, and his son, both with Gardner syndrome, were noted to have multiple pilomatrixomas. The father's earlier diagnosis of Gardner syndrome was crucial for identifying the genetic condition in his son. This case underscores the importance of recognizing familial skin lesions as potential indicators of Gardner syndrome [30].

Gastric Adenocarcinoma

Gastric adenocarcinoma is a severe and heterogeneous malignant disease influenced by multiple genetic and environmental factors [31]. It originates from the glandular cells of the gastric mucosa and begins with atrophy, which can sometimes progress to atypical hyperplasia or intraepithelial neoplasia [32]. It can appear in different forms, such as intestinal, diffuse and mixed types, which are categorized according to the Lauren and WHO classification systems [33]. Gastric adenocarcinoma is the most common histological type of gastric cancer and the third most common cause of cancer-related deaths globally [34,35]. Metastatic lesions to the skin from internal cancers are relatively uncommon but can occasionally be the first manifestation of underlying malignancies, typically arising from cancers of the breast, lung or large bowel. Clinically, they often appear at a more advanced stage of cancer and are associated with a poor prognosis [36]. Most cases of cutaneous metastasis from gastric cancer manifest with an emerged nodule or erysipelas-like skin lesions. In patients with local metastases, the resection of a cutaneous lesion may be helpful [37].

Some dermatological conditions, although rare and nonspecific, are related to gastric adenocarcinoma and may potentially serve as paraneoplastic markers. The Leser-Trélat sign means the sudden eruption of multiple seborrheic keratoses. It is characteristic of this sign, which is considered a potential cutaneous marker of malignancy, such as gastric cancer [38]. Another condition, acanthosis nigricans condition, is characterized by velvety, hyperpigmented skin affecting mostly the axillary and neck areas [39]. It is rarely indicative of some kind of internal malignancy called malignant acanthosis nigricans, often associated with GI malignancies [40]. Additionally, tripe palms are a very uncommon paraneoplastic syndrome characterized by thickened, velvety and wrinkled skin on the palms, occurring mostly due to gastric cancer and other malignancies [41]. For gastric adenocarcinoma, cutaneous metastases and paraneoplastic syndromes can provide early clues to an otherwise asymptomatic malignancy. A clinical case reported a 49-year-old woman presented with cutaneous metastases as the initial manifestation of gastric adenocarcinoma. Multiple skin nodules were observed, which, upon biopsy, proved to be metastatic lesions. Her disease was at an advanced stage despite systemic chemotherapy [42].

Pancreatic Malignancy

Early diagnosis of pancreatic cancer remains challenging due to its non-specific symptoms. However, emerging evidence suggests that dermatological manifestations may serve as warning signs. A study emphasized the importance of pancreatic panniculitis, which, when associated with malignancy, confers a poor prognosis. Panniculitis, an inflammatory disorder of the subcutaneous fat, is classified into septal panniculitis or lobular panniculitis. Pancreatic panniculitis is a type of lobular panniculitis [43]. The manifestations of panniculitis include erythematous and painful subcutaneous nodules that may ulcerate, with brown-colored, viscous to oily fluid exiting the ulcer as a sign of colliquative fat necrosis; it is most commonly localized in the distal parts of the lower limb, especially around the knee joint [44]. Pancreatic panniculitis occurs in approximately 2-3% of all patients with pancreatic disease. It is most commonly associated with acute and chronic pancreatitis and acinar cell carcinoma of the pancreas but is rarely associated with pancreatic ductal adenocarcinoma [43]. Glucagonoma syndrome, a type of NET, characterized by overproduction of glucagon, manifests as diabetes mellitus, stomatitis, anemia, weight loss, and the skin condition necrolytic migratory erythema. Necrolytic migratory erythema is manifested by the formation of induced lesions with crusts on the periphery, the development of such lesions is rapid, in which in a few days, the erythematous foci change into necrolytic foci with crusts and subsequent hyperpigmentation [44]. This emphasizes the role of dermatologists in identifying those skin manifestations.

Regular dermatological screening is suggested for GI neoplasms. Patients with a known history of GI neoplasm should undergo regular dermatological evaluations to monitor for skin changes and lesions. Thus, a multidisciplinary approach involving dermatologists, oncologists and geneticists may be beneficial for disease management and outcome optimization. Continuous research and clinical awareness are crucial for improving our understanding and treatment of these diseases. Dermatological manifestations are vital for early diagnosis and management. Identifying and treating such cutaneous signs promptly can lead to early intervention and potentially better outcomes for patients. All conditions have been summarized in Table 1.

**Table 1:** Summary of dermatological manifestations of primary gastrointestinal neoplasms

Primary Gastrointestinal Neoplastic Condition	Associated Dermatological Manifestations	Description
Pancreatic Cancer [43,44]	Pancreatic Panniculitis	Erythematous, painful subcutaneous nodules, often in the distal lower limbs. May ulcerate and release viscous brown fluid. Associated with poor prognosis.
	Necrolytic Migratory Erythema	Rapidly evolving erythematous lesions with crusts and hyperpigmentation. Frequently linked to glucagonoma syndrome.
Muir-Torre Syndrome [19]	Sebaceous Tumors	Tumors of the sebaceous glands, such as sebaceous adenomas and carcinomas. Often the first signs of Muir-Torre syndrome.
	Keratoacanthomas	Skin tumors that may mimic squamous cell carcinoma. Appear as a single, dome-shaped lesion.
Carcinoid Syndrome [20-24]	Necrolytic Migratory Erythema	Similar to the manifestation seen in glucagonoma syndrome; characterized by crusting and hyperpigmentation
	Flushing	Episodes of facial redness and warmth. Common in carcinoid syndrome.
	Pellagra-Like Symptoms	Skin manifestations resembling pellagra, including dermatitis, often due to vitamin deficiencies.
	Scleroderma-Like Features	Skin thickening and tightening without Raynaud’s phenomenon
Peutz-Jeghers Syndrome [2,12,13]	Melanotic Macules	Hyperpigmented macules typically on the lips, palate, and tongue. Often presents before gastrointestinal symptoms.
Cowden Syndrome [14-18]	Trichilemmomas	Benign tumors of the hair follicle, typically appearing on the face.
	Oral Papillomas	Small, benign growths in the oral cavity, including the buccal mucosa and tongue.
	Acral Keratosis	Thickened, rough patches on palms, soles, and elbows.
	Xanthomas	Yellowish skin lesions, often associated with lipid disorders.
	Vitiligo	Loss of skin pigmentation leading to white patches
	Palmoplantar Keratosis	Thickened, rough skin on the palms and soles, sometimes presenting with hyperkeratosis.
Gastric Adenocarcinoma [32-42]	Leser-Trélat Sign	Sudden eruption of multiple seborrheic keratoses, potentially indicative of malignancy.
	Acanthosis Nigricans	Velvety, hyperpigmented skin often in the axillary and neck areas; can indicate malignant acanthosis nigricans.
	Tripe Palms	Thickened, velvety, and wrinkled skin on the palms; associated with gastric cancer and other malignancies.
Gardner Syndrome [25-30]	Epidermoid Cysts	Common cystic lesions typically on the face, scalp, and trunk.
	Dermatofibromas	Benign nodules usually found on the extremities and trunk.
	Desmoid Tumors	Aggressive, benign tumors often located in the abdominal wall; can be locally invasive.
	Osteomas	Benign bone tumors typically affecting the mandible and skull; often asymptomatic.

Autoimmune diseases

Autoimmune diseases are a diverse group of disorders characterized by an abnormal immune response directed against the body's own tissues. These conditions can have a wide range of manifestations, including dermatological changes, which can serve as important diagnostic and prognostic indicators.

Autoimmune Liver Disorders

Autoimmune conditions of the liver such as primary biliary cirrhosis (PBC), primary sclerosing cholangitis (PSC) and autoimmune hepatitis are a few that have been associated with dermatological manifestations. PBC is an autoimmune disease in which intrahepatic destruction of bile ducts leads to cholestasis and is more commonly seen in women. The anti-mitochondrial antibody is found to be very specific for PBC. PSC is a chronic autoinflammatory disease of both intrahepatic and extrahepatic bile ducts. It presents with pruritis and right upper quadrant pain. Laboratory tests show elevated alkaline phosphatase and gamma-glutamyltransferase. Autoimmune hepatitis is a rare autoimmune disorder that ranges from transaminitis to acute liver failure. It is diagnosed based on the presence of autoantibodies and liver biopsy [45].

Xanthomas

Xanthomas are a commonly seen finding in PBC. They are localized depositions of lipids in an organ system, which occur due to the permeation of lipoproteins through the blood vessel into tissues like skin and tendons. Seventy-five percent of patients with PBC have dyslipidemia due to accumulation of lipoprotein X and this leads to the development of xanthomas [46]. They are the first presenting finding in PBC in less than 1% of patients and are commonly seen on knees, elbows, neck, trunk and breasts [47]. In patients with PBC, xanthomas did not resolve with the usual treatment of ursodeoxycholic acid. In a case report of a 33-year-old woman with xanthomas, plasmapheresis led to a slight reduction of the lesions but notable regression was seen only five months after a liver transplant [46].

Morphea

Morphea is a localized form of scleroderma which primarily affects the skin. It can be generalized subcutaneous, guttate, nodular and linear in form. According to a study done to determine the association between PBC and morphea, it was found that there was a significant association with an odds ratio of 8.3 (p<0.0001) [48]. A study reported that in a 27-year-old male patient generalized morphea was found to be linked to autoimmune hepatitis. Upon confirmation of liver biopsy, the patient was treated with 200mg intravenous methylprednisolone, which led to the complete resolution of morphea. The trunk and extremities were the most commonly observed sites of involvement. It was also found that generalized morphea was more commonly linked to autoimmune disorders when compared to linear or plaque morphea [49].

Psoriasis

Psoriasis is a chronic autoimmune disorder with a genetic predisposition which presents. The disease usually occurs in flares with scaly, erythematous patches with symptom-free intervals. The flares could be caused by a triggering event like a drug or infection. The plaques in psoriasis are commonly seen on elbows, knees and scalp [50]. It is found to be associated with a number of autoimmune liver conditions like PBC, PSC and autoimmune hepatitis. It was found that PBC was significantly associated with psoriasis with an odds ratio of 2.4 (p<0.0001) [48]. PSC is also found to coexist with psoriasis due to the involvement of similar human leukocyte antigen (HLA) foci. Psoriasis is also found to be linked to autoimmune hepatitis because interleukin-17 (IL-17) was found to be a common key player in the pathogenesis of both diseases in a study performed on mice. Psoriasis can be triggered by infections, stress, smoking and alcohol consumption. Because there are higher chances of psoriasis developing in PBC, patients should be screened for psoriasis. It was also noticed that psoriatic eruptions may sometimes be seen before the development of PBC symptoms, the cause of which could be PBC being a slowly progressive disease. Therefore, it is advised that patients with symptoms of psoriasis should be frequently monitored with liver function tests. It has been noted that the standard treatment for PBC with ursodeoxycholic acid has alleviated symptoms of psoriasis [51].

Systemic Sclerosis (SS)

SS is an autoimmune condition in which fibrosis of the skin and internal organs is seen. It can be divided into limited and diffuse forms based on the extent of involvement of the organs and skin. Limited form involves the cutaneous sclerosis of fingers, hands and face, which later spreads to the center of the body. In the diffuse form, the heart, lung and kidney can be involved early on and is considered a more life-threatening form. SS has been linked to autoimmune liver conditions like PBC and autoimmune hepatitis. The prevalence of SS in PBC ranges from 1.4% to 12.3%, and 93% of the reported cases were of the limited SS subtype. Anti-centromere antibody, which is the most common antibody seen in limited SS, is also found in 9-30% of patients with PBC [52]. A rare case of SS associated with cirrhosis due to autoimmune hepatitis was reported in a 51-year-old female patient. According to the case report, the patient had fulfilled the criteria for autoimmune hepatitis by the International Autoimmune Hepatitis group and also had a total score of 15 for the classification of SS. The above studies show that there should be a high degree of suspicion of the development of autoimmune liver disease in a patient with preexisting SS and therefore, liver function tests should be done as frequently as possible to treat the liver condition. Treatment of SS with liver conditions is usually managed by treatment specific to the symptoms manifesting. It was advised to use high-dose corticosteroids with caution in autoimmune hepatitis as it can precipitate scleroderma renal crisis in patients with SS [53].

Celiac Disease (CD)

CD is an immune systemic disorder caused by an abnormal response to gluten and related genetically primarily to those with specific HLA genes; studies confirmed that every patient with dermatitis herpetiformis (DH) and CD had the alleles HLA-DQ2 or HLA-DQ8. The etiopathogenesis is complex and only partially understood; both disorders involve a complicated interaction between environmental and genetic factors triggered by gluten ingestion from wheat, rye, and barley - primarily gliadin - in genetically predisposed individuals. This interaction results in an inflammatory cascade leading to the production of circulating IgA and IgG autoantibodies targeting transglutaminase (TG). TG2 is a multifunctional tissue protein highly expressed in basal keratinocytes, dermal capillaries, as well as blood vessel walls and small bowel, while TG3 is found in the epidermis, esophagus, brain, the eyes and lowly expressed in the small intestine [54-59].

DH and CD are linked with specific HLAs including HLA-DQ2, HLA-DQ8, HLA-DR3, HLA-A1 and HLA-B8. The prevalence of these antigens in DH and CD is notably high, HLA-B8 is found in 58-90% of patients, HLA-DR3 in 88-95%, and HLA-DQ2 in 95-100%, compared to their occurrence in healthy individuals [60]. About 85% of Caucasian patients with DH have HLA-DQ2, while most of the remaining 15% have HLA-DQ8. Around 25-30% of the patients have preserved villous architecture without or with an increased number of intraepithelial lymphocytes and crypt hyperplasia, but in up to 70-75% of DH patients moderate to severe villous atrophy with crypt hyperplasia is present at diagnosis [54].

DH is associated with CD-sharing antibodies against tissue TG and is thought to be due to cross-reaction of anti-tTG IgA against tissue TG, this process generates a more vigorous CD4+ T-helper cell activation, which can result in intestinal mucosa inflammation, symptoms of malabsorption, and secondary extra-intestinal manifestations such as DH. It’s known that TG3, an enzyme belonging to the same family of TG2 but expressed above all in the epidermis, was the main autoantigen of DH. One hypothesis concerns an epitope between TG2 and TG3 shares a high sequence homology [59,61].

DH affects about 10-13% of CD patients in Europe and North America, other studies show a prevalence of about 17-20% of DH between untreated CD. The highest prevalence of DH has been observed in Finland, with approximately 75 cases per 100,000 people; it is mostly diagnosed in adulthood and can also occur in childhood and adolescence. Studies on DH have reported a male-to-female ratio ranging from 1.1 to 1.9. As evidenced by a cross-sectional study indicated that the most common skin manifestation was DH (16.0%), predominantly observed in males (18.9%) [62]. While CD generally shows a higher prevalence in females [56,63,64].

A study conducted revealed that 60% of patients with DH had a silent CD with no intestinal symptoms, whereas 10-20% had classic intestinal symptoms and another 20% had atypical symptoms, Reunala et al. described dermatitis herpetiform as the most common extraintestinal manifestation of CD. A study conducted in Finland on 18,538 CD patients found that 17% (3,121 individuals) also had associated DH [62]. The current estimations of the incidence of DH in CD are one in every eight patients [58]. In general, DH is a rare disease diagnosed in 11.2 to 75.3 per 100,000 people in the United States and Europe with an incidence of 0.4 to 3.5 per 100,000 people per year [59,61]. Whereas DH can manifest solely in the skin characterized by an extremely pruritic or burning sensation on the skin, small vesicles and papules mostly affect elbows, knees and buttocks that may be obscured by excoriations [64,65]. Most patients have some degree of histologic features of CD in their small bowel, although it is generally less severe than in CD, patients might experience GI symptoms like bloating, diarrhea or constipation, but these symptoms are typically mild if they occur [66]. Diagnosis before 16 years of age is rare and the highest incidence is among men between 60 and 69 years old and women between 50 and 59 years old [64].

IBD

IBD comprises Crohn's disease and UC and is primarily an inflammatory condition of the GI tract but can have extra-intestinal manifestations, including dermatological conditions. The skin and the gut are connected through the gut-skin axis, a concept that highlights the interaction between the GI and immune systems and their impact on skin health. Dysbiosis in the gut can lead to systemic inflammation, which in turn can manifest in the skin and can vary widely in presentation and severity. Many dermatological conditions associated with IBD are also autoimmune in nature, sharing common pathogenic mechanisms involving immune dysregulation and chronic inflammation [67,68].

**Figure 1:**
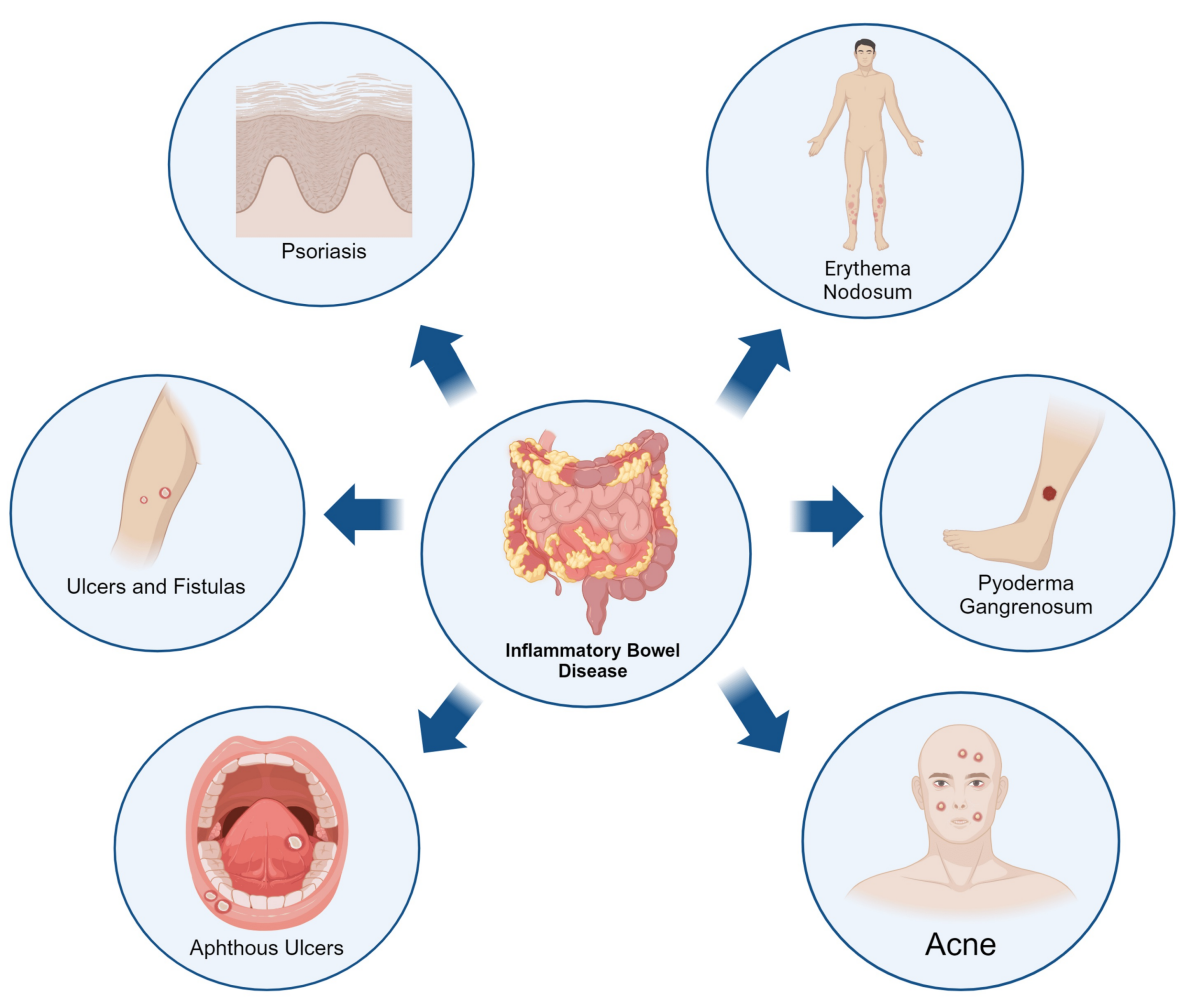
Skin manifestations of inflammatory bowel disease Figure made using biorender.com.

The pathophysiology underlying these dermatological manifestations often involves complex immunological interactions. For instance, immune cells activated in the gut can migrate to the skin, leading to inflammation and characteristic skin lesions [68]. Additionally, cytokines and other inflammatory mediators including IL-1 and 6 and tumor necrosis factor (IL-1, IL-6, TNF) produced in response to intestinal inflammation can affect the skin, exacerbating existing conditions or triggering new dermatological issues. Shared genetic predispositions and environmental triggers may also play a role in the development of both IBD and associated skin disorders. Some medications used to treat Crohn's disease and UC, such as corticosteroids and immunosuppressants, can cause skin side effects or worsen existing skin conditions. About 15 to 40% of the patients suffering from IBD present with dermatological symptoms. While estimates vary depending on the population studied and specific skin conditions considered, it's generally accepted that dermatological manifestations occur in around 5-20% of individuals with UC and 20 to 40% of patients with CD [1,69-71]. This interconnection suggests that effective management of Crohn's disease requires a comprehensive approach that addresses both GI and dermatological symptoms (Figure 1).

EN

EN is characterized by the sudden onset of tender, red nodules, typically located on the shins [72]. It is the most common dermatological manifestation of IBD, occurring in approximately 15% of patients [73]. The pathophysiology of EN involves an inflammatory reaction in the subcutaneous fat, potentially linked to an immune complex-mediated vasculitis [74]. The presence of EN often correlates with flares of UC and CD activity, particularly involving the intestines and usually resolves as the underlying inflammation is controlled [75]. This association underscores the importance of monitoring patients with EN for possible exacerbations of IBD, as the skin condition can serve as a clinical indicator of underlying GI inflammation [76].

PG

PG usually presents with painful pustules or nodules that break down and will later develop into expanding ulcers with irregular borders. PG is usually located on the extensor side of the lower limbs. This condition occurs in approximately 0.7% of all patients with Crohn's disease [77]. The pathophysiology in PG is not yet fully understood but it is thought to be an abnormal immune response, which could affect neutrophil dysfunction. The treatment with this condition can be highly frustrating for the patients because of its painful presentation and difficult approach. The treatment usually consists of therapy with immunosuppressant drugs including corticosteroids and cyclosporine A [78,79].

Cutaneous Crohn’s Disease

Cutaneous Crohn's disease is a granulomatous lesion that can affect any part of the skin, but it usually affects the perineum and perianal regions. Due to this lesion’s location, it can be quite painful and limiting for the patients functionally. Cutaneous lesions usually provide a cue to more extensive disease that may not easily respond to medical management and in consequence it usually requires surgical intervention [77].

Psoriasis and Psoriasiform Dermatitis

The prevalence of psoriasis and psoriasiform dermatitis is increased in patients with Crohn's disease and UC as compared to healthy individuals. It has been hypothesized by several studies that this correlation between the appearance of psoriasis and psoriasiform dermatitis in patients with IBD is probably caused by a similar genetic and immunological pathway between the diseases. The presentation of these lesions is usually as erythematous plaques covered by silvery scales. Due to these pathophysiological similarities, the development of targeted therapies that address not only IBD but also its dermatologic manifestations such as psoriasis is an area of frequent research [80-82].

Epidermolysis Bullosa Acquisita (EBA)

EBA is an autoimmune blistering disorder associated with subepidermal blisters and erosions that can manifest even during infancy. This is not a common dermatological manifestation of IBD; however, it has been linked with patients with Crohn's disease. The most common autoantibodies present in EBA are against type VII collagen, which in consequence results in skin fragility and blister formation [83,84].

Fistulizing Skin Metastases

Fistulizing cutaneous metastases is a dermatological condition that has been associated with Crohn's disease. This condition is an extremely rare dermatological manifestation of Crohn's disease. However, it's a serious condition that warrants immediate medical attention. This condition is characterized by abnormal communication between the skin and other tissues. The treatment of the common ulcerative lesions in appearance may be quite challenging most of the time. These lesions usually are a good indicator of the severity of systemic involvement in Crohn's disease [85].

Pediatric Cutaneous Crohn’s Disease

The median age of presentation of pediatric cutaneous Crohn's disease in males is 10.4 years of age and in females is 11.2 years of age. The most common site of cutaneous Crohn's disease in children's manifestations is in the genitals, constituting 75.3% of all lesions. The second most common site of cutaneous Crohn's disease manifestations is in the perianal area with 55.1% of the patients presenting lesions in this area. The most commonly prescribed treatments for these lesions in the pediatric population are oral corticosteroids and metronidazole [86,87].

Hidradenitis Suppurativa (HS)

HS is a chronic recurrent inflammatory dermatological condition that presents with debilitating nodules, abscesses and sinus tract formations. Although HS is a rare manifestation of GI diseases, it presents in about 0.3 to 4% of patients suffering from CD. Studies have reflected that the probability of developing HS is nine times higher in patients with Crohn's disease as compared to normal individuals. A strong correlation in pathophysiology related to IL-1 and IL-6 and TNF has been established between the two conditions and supplementation of anti-TNF therapy in Crohn's disease patients has resulted in the resolution of HS symptoms [88-90].

The dermatological manifestations of IBD are diverse and can provide important clues to the diagnosis and management of this complex condition. Understanding these skin manifestations can aid in early detection and prompt treatment of Crohn's disease and UC, improving overall patient outcomes. By recognizing the intricate connection between the gut and skin, healthcare providers can develop more comprehensive and effective treatment strategies for patients with IBD [69-71]. All findings have been summarized in Table 2.

**Table 2:** Summary of dermatological manifestations in primary autoimmune gastrointestinal diseases

Primary Autoimmune Gastrointestinal Condition	Autoimmune Liver Diseases: Primary Biliary Cholangitis, Primary Sclerosing Cholangitis and Autoimmune Hepatitis [45-53]	Celiac Disease [54]	Inflammatory Bowel Disease: Crohn’s Disease and Ulcerative Colitis [68-90]
Dermatological Manifestations	Xanthomas, Morphea, Psoriasis and Systemic Sclerosis	Dermatitis Herpetiformis	Erythema Nodosum, Pyoderma Gangrenosum, Psoriasis, Cutaneous metastasis and Epidermolysis Bulla Acquisita

## Conclusions

GI diseases can present with multiple extra-intestinal manifestations, and dermatological symptoms are among the most prevalent manifestations. Dermatologic signs and symptoms that correspond to autoimmune and neoplastic GI pathologies promote the detection of a multitude of diseases at an early stage and also aid in understanding the disease progression. Clinicians, especially dermatologists, should learn to recognize these dermatological signs related to occult GI pathologies and manage patients accordingly as per clinical suspicion.

A multidisciplinary approach is important in the diagnosis and management of systemic diseases because it highlights the complicated relationship between GI disorders and dermatological manifestations. Developing a deeper understanding of the genetic and pathogenic links between dermatological and GI diseases will enable the medical community to devise better diagnostic and therapeutic strategies. It is imperative to establish diagnostic criteria to refer patients in a timely manner for appropriate diagnostic workup and management.
